# Proinsulin Atypical Maturation and Disposal Induces Extensive Defects in Mouse *Ins2^+/Akita^* β-Cells

**DOI:** 10.1371/journal.pone.0035098

**Published:** 2012-04-03

**Authors:** Qingxin Yuan, Wei Tang, Xiaoping Zhang, Jack A. Hinson, Chao Liu, Kwame Osei, Jie Wang

**Affiliations:** 1 Division of Endocrinology, Diabetes, and Metabolism, Department of Internal Medicine, The Ohio State University, Columbus, Ohio, United States of America; 2 Department of Pharmacology and Toxicology, University of Arkansas for Medical Sciences, Little Rock, Arkansas, United States of America; 3 Division of Endocrinology, Jiangsu Province Hospital on Integration of Chinese and Western Medicine, Nanjing University of Chinese and Western Medicine, Nanjing, Jiangsu, China; Consejo Superior de Investigaciones Cientificas, Spain

## Abstract

Because of its low relative folding rate and plentiful manufacture in β-cells, proinsulin maintains a homeostatic balance of natively and plentiful non-natively folded states (i.e., proinsulin homeostasis, PIHO) through the integration of maturation and disposal processes. PIHO is susceptible to genetic and environmental influences, and its disorder has been critically linked to defects in β-cells in diabetes. To explore this hypothesis, we performed polymerase chain reaction (PCR), metabolic-labeling, immunoblotting, and histological studies to clarify what defects result from primary disorder of PIHO in model *Ins2^+/Akita^* β-cells. We used T antigen-transformed *Ins2^+/Akita^* and control *Ins2^+/+^* β-cells established from Akita and wild-type littermate mice. In *Ins2^+/Akita^* β-cells, we found no apparent defect at the transcriptional and translational levels to contribute to reduced cellular content of insulin and its precursor and secreted insulin. Glucose response remained normal in proinsulin biosynthesis but was impaired for insulin secretion. The size and number of mature insulin granules were reduced, but the size/number of endoplasmic reticulum, Golgi, mitochondrion, and lysosome organelles and vacuoles were expanded/increased. Moreover, cell death increased, and severe oxidative stress, which manifested as increased reactive oxygen species, thioredoxin-interacting protein, and protein tyrosine nitration, occurred in *Ins2^+/Akita^* β-cells and/or islets. These data show the first clear evidence that primary PIHO imbalance induces severe oxidative stress and impairs glucose-stimulated insulin release and β-cell survival as well as producing other toxic consequences. The defects disclosed/clarified in model *Ins2^+/Akita^* β-cells further support a role of the genetic and stress-susceptible PIHO disorder in β-cell failure and diabetes.

## Introduction

Pancreatic β-cell failure in diabetes is characterized primarily by progressive loss of insulin production and β-cell mass. β-cell failure has been attributed to autoimmune assault in type 1 diabetes and to glucolipotoxicity, amyloid deposition, insulin resistance, and endoplasmic reticulum (ER) and/or oxidative stress in type 2 diabetes [1]–[10]. However, the intrinsic mechanisms underlying β-cell susceptibility to stress and damage remains largely unclear. Are β-cells overwhelmed by such stress and its associated toxic byproducts, such as reactive oxygen species (ROS), or does the attenuation of beneficial genes and/or trigger of harmful genes prove detrimental to some inherent property specific to cell type? Data exist to support both views, but β-cell dysfunction and damage resulting from various stress insults, such as from hypoglycemia/hypoxia [11], [12] and chronic hyperglycemic and hyperlipidemic conditions [2]–[10], [13]–[15], in addition to influences by genetic variations support the second view. As well, low expression levels of genes protective against oxidative stress or hypoglycemia/hypoxia insult, such as catalase or hypoglycemia/hypoxia inducible mitochondrial protein 1, are implicated in the susceptibility of β-cells to stress [16], [17].

Moreover, as β-cells mature to produce insulin, they become sensitive to cytokine insult [18]. Insulin is the most abundant and unique protein produced in β-cells and constitutes up to 14% of the dry weight of rodent islets or β-cells [19], [20]. Studies of protein biosynthesis in rodent/carp islets have shown incorporation of 6 to 30% of radioactive amino acids into preproinsulin in basal or glucose-stimulated conditions [21], [22], although many proteins are produced in islets/β-cells. Proinsulin is the precursor of most abundant insulin in the ER, preserves a low relative folding rate, and bears the greatest burden in the protein folding of β-cells [23]. Thus, it maintains a homeostatic balance of natively and plentiful non-natively folded states (i.e., proinsulin homeostasis, PIHO) in β-cells as a result of the integration of maturation and disposal processes for adaptation [23]. Both the substrate-favored lysosomal and proteasomal pathways participate in the normal maintenance of PIHO in β-cells [24]. The contrast of low relative folding rate with plentiful amounts of insulin precursor manufactured in β-cells renders PIHO susceptible to genetic and environmental influences, such as changes in cellular energy and Ca^2+^, ER or oxidative stress, and cytokine insults [23], [24]. PIHO disorders have been found in *Ins2^+/Akita^* [23], non-obese diabetic (NOD), and db/db mice (unpublished data), unique models that represent the primary forms of diabetes in humans. Monogenic disorders include syndromes characterized in early studies that resemble type 2 diabetes caused by mutations in the insulin gene in non-neonatal patients [25]. Recently, up to 20% of human neonatal diabetes with proband variants for type 1, and even type 2, has been associated with defective insulin genes and demonstrated pathogenesis resembling that in the *Ins2^+/Akita^* mouse model [26]–[29]. Those subjects with defects in the same preproinsulin molecule showed β-cell failure resembling that in general diabetes. Therefore, we have suggested that disorder in PIHO is critically linked to β-cell failure and diabetes [23].

To test this hypothesis, we further characterized defects that resulted from primary PIHO in *Ins2^+/Akita^* β-cells derived from the diabetic Akita mouse [30]. In the Akita mouse model, diabetes occurs early, at age 4 weeks, as a result of primary PIHO disorder induced by a point mutation (*Ins2^+/Akita^*, C96Y) in an allele of the insulin 2 gene [23], [31]. Because it is difficult to achieve sufficient numbers of β-cells/islets from diabetic *Ins2^+/Akita^* mice for study, we evaluated PIHO disorders and their consequences using T antigen-transformed *Ins2^+/Akita^* and control *Ins2^+/+^* β-cells established from Akita and wild-type littermate mice [30]. Similar findings of disorders in PIHO, subsequent proinsulin conversion, and enhanced response to ER stress between the established *Ins2^+/Akita^* β-cell lines and the islets of *Ins2^+/Akita^* mice [23], [30]–[32] demonstrate the utility of *Ins2^+/Akita^* and control *Ins2^+/+^* β-cell lines for such study. However, other typical defects of β-cells with known association to diabetes of general forms remain uncharacterized in this model of β-cell dysfunction; these include oxidative stress and abnormalities in glucose-stimulated insulin secretion (GSIS) and β-cell survival. Unlike islets exposed to *in vivo* hyperglycemic or euglycemic environments in diabetic or non-diabetic subjects, the *Ins2^+/Akita^* and *Ins2^+/+^*β-cell lines remain in the customary culture condition (with 25.5 mmol/L glucose) in which they are established [30] and are therefore ideal models for testing our hypothesis. In this follow-up study using the dysfunctional *Ins2^+/Akita^* β-cell model, we further disclose/clarify the toxic consequences of PIHO disorder that are generally associated with diabetes.

## Results

### No apparent transcriptional and translational defect contributes to the reduced proinsulin and insulin content of *Ins2^+/Akita^* β-cells

**Figure 1:**
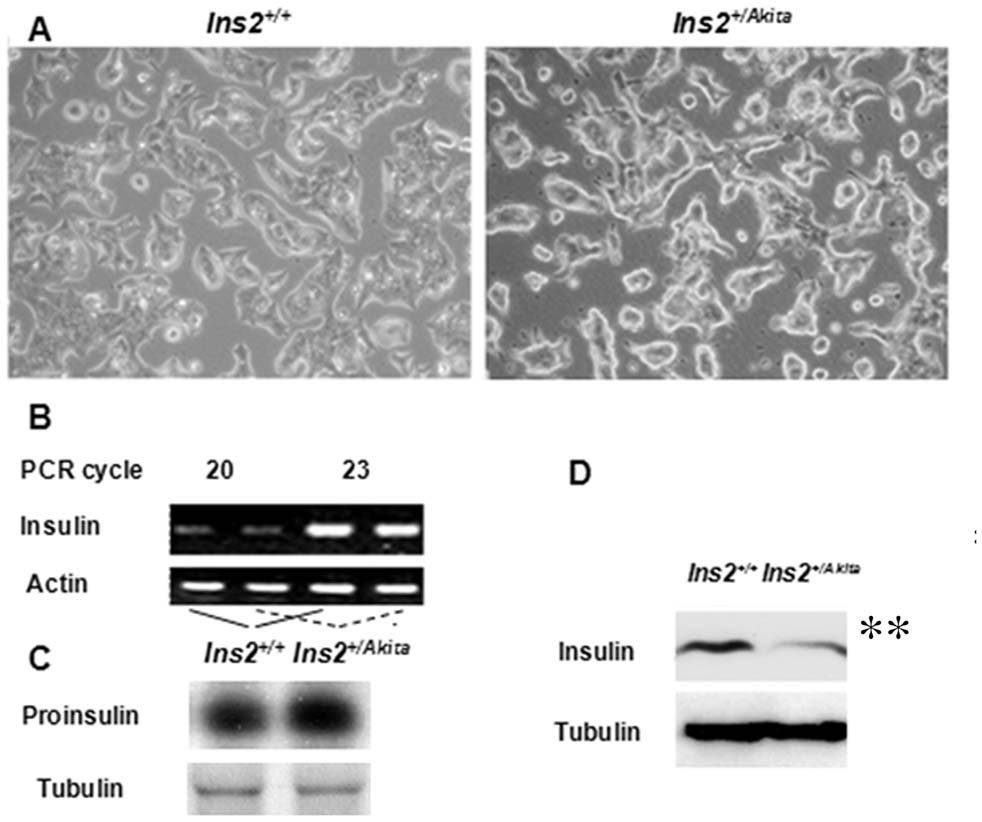
No apparent transcriptional and translational defect contributes to the reduced proinsulin and insulin content of *Ins2^+/Akita^* β-cells. (A) Light microscopic images (with 200-fold amplifications) of *Ins2^+/Akita^* and *Ins2^+/+^* β-cells cultured for 48 hours under customary condition (containing 25. 5 mmol/L glucose). (B) We examined the levels of insulin transcripts in *Ins2^+/Akita^* and control *Ins2^+/+^*β-cells using quantitative PCR approaches described in Materials and Methods. (C) Newly synthesized ^35^S-proinsulin or^35^S- tubulin in *Ins2^+/Akita^* or *Ins2^+/+^*β-cells during a 30-minute labeling course was immunopurified with C-peptide or tubulin antisera and dissolved by reduced SDS-PAGE for autoradiography. (D) We used immunoblot analysis to evaluate the levels of immunoreactive proinsulin and insulin of *Ins2^+/Akita^* and *Ins2^+/+^*β-cells cultured for 48 hours under customary condition. Data shown in (A to D) represent 3 independent experiments.

In model *Ins2^+/Akita^* β-cells/islets, the consistent reduction in proinsulin and insulin content and secreted insulin has been attributed to defects in transcriptional [30], translational [7], or post-translational [31], [33] regulation in addition to β-cell depletion. To clarify the reason for this reduction, we performed studies in the established *Ins2^+/Akita^* and *Ins2^+/+^*β-cells cultured under the customary high (25. 5 mmol/L) glucose condition. In light microscopic images (with 200-fold amplifications), the only apparent difference in morphology between these 2 cell lines was a seemingly slight hypertrophy of *Ins2^+/Akita^* β-cells (Fig. 1A). Using approaches described previously [17], [23], [31], our quantitative polymerase chain reaction (PCR) and metabolic-labeling analyses detected no decline in the transcriptional and translational levels of proinsulin in *Ins2^+/Akita^* β-cells versus control *Ins2^+/+^* β-cells (Fig. 1B and 1C; transcript, 103.8±8.2% versus 100.0±6.5%; ^35^S-proinsulin, 109.7±6.3% versus 100.0±7.2%; *P*>0.05, n = 3). Neither did microarray analysis detect any reduction in the insulin transcripts of *Ins2^+/Akita^* β-cells (unpublished observations). These findings indicate that abnormalities responsible for the reduced insulin content in *Ins2^+/Akita^* versus *Ins2^+/+^* β-cells (Fig. 1D; 27.9±4.3% versus 100.0±8.1%; *P*<0.01, n = 3) and the reduced insulin secretion [17], [23], [31], [32] occur mainly in the regulation of post-translational processing.

### Glucose response remained normal in proinsulin biosynthesis but was impaired for insulin secretion of *Ins2^+/Akita^* β-cells

**Figure 2:**
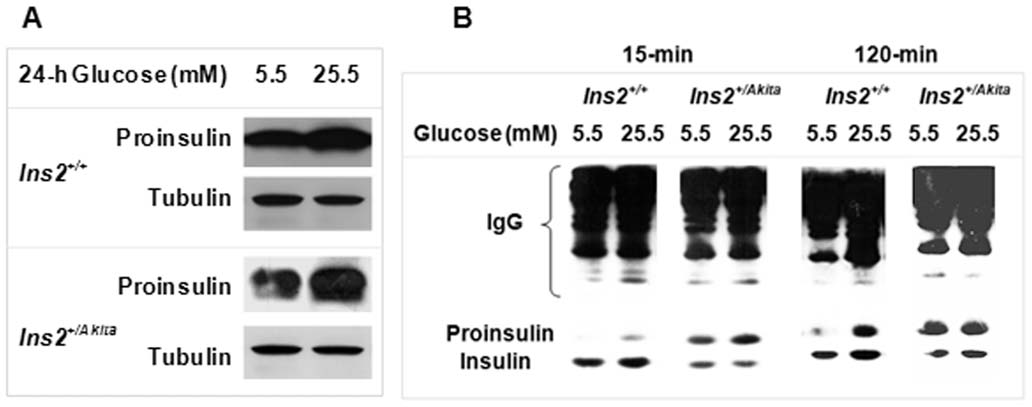
Glucose response remained normal in proinsulin biosynthesis but was impaired for insulin secretion of *Ins2^+/Akita^* β-cells. (A) We examined the levels of proinsulin in *Ins2^+/Akita^* and *Ins2^+/+^* β-cells cultured for 24 hours at 5.5 or 25.5 mmol/L glucose conditions by C-peptide immunoblot. (B) Proinsulin and insulin secreted by *Ins2^+/Akita^* or *Ins2^+/+^* β-cells during a 15- or 120-minute course of glucose stimulation were immunopurified with insulin and C-peptide antisera, resolved by 16.5% tricine non-reduced SDS-PAGE, and examined by insulin immunoblot. Data shown in (A and B) represent 3 independent experiments.

Impaired glucose-stimulated insulin secretion in diabetes is well recognized, but its underlying mechanisms remain largely unclear [34]–[36]. To explore the association between early PIHO disorders and subsequent GSIS abnormalities, we examined the response in *Ins2^+/Akita^* and *Ins2^+/+^* β-cells of proinsulin biosynthesis or insulin secretion to stimulation in a culture with high glucose concentration (25.5 mmol/L) after one-day preculturing with 5.5 mmol/L glucose. Immunoblot analysis showed similar increases in the amount of proinsulin in *Ins2^+/Akita^* β-cells and control β-cells in response to 24-hour stimulation in the high glucose environment (Fig. 2A, 50.5±7.3% versus 54.3±11.1%; *P*>0.05, n = 3), findings suggesting that there was no apparent defect in the regulation of proinsulin transcriptional and translational responses to glucose. However, following either a 15- or 120-minute course of glucose stimulation, the amount of insulin secreted by *Ins2^+/Akita^* β-cells was reduced compared with that of control *Ins2^+/+^* β-cells (Fig. 2B; 15 minutes, 53.5±12.7% versus 100.0±16.0%; 120 minutes, 58.8±27.8% versus 100.0±36.0%; *P*<0.05, n = 3). The release of insulin during the 15- or 120-minute course of glucose stimulation mimicked that in the first or second phase of GSIS by islets *in vivo*. These results demonstrate the impairment of GSIS in *Ins2^+/Akita^* β-cells as a result of PIHO disorder. Moreover, the finding of an abnormally high ratio of proinsulin/insulin in the secreted proinsulin and insulin from *Ins2^+/Akita^* β-cells (Fig. 2B) was consistent with our recently reported findings [32].

As shown in Fig. 2B, our immunoprecipitation approach with mixed antisera to C-peptide and insulin pulled down large amounts of secreted proinsulin and insulin from culture media. Immunoblot analysis of these secreted proinsulin and insulin allowed us to find the above described impairments in the insulin secretion of *Ins2^+/Akita^* β-cells versus control *Ins2^+/+^* β-cells. Our immunoprecipitation approach with mixed antisera to C-peptide and insulin showed an advantage in recovery of proinsulin compared to the immunoprecipitation approach customarily using insulin antisera alone (Fig. S1). These outcomes clearly demonstrated that the immunoprecipitation combined with the immunoblot approach that we used in this study and reported previously [32] is a reliable procedure in addition to the traditional radioimmunoassay approach in evaluation of GSIS and its impairments. Furthermore, this method can evaluate the proportional composition of secreted insulin and proinsulin when insulin in secreted proteins is measured at the same time. This is because proinsulin as a component of insulin immunoreactive materials, which cannot be well distinguished or determined by radioimmunoassay using insulin antibody alone, was indeed detected and distinguished by using the immunoprecipitation combined with the immunoblot method shown in our study.

### Structural abnormalities of *Ins2^+/Akita^* β-cells

**Figure 3:**
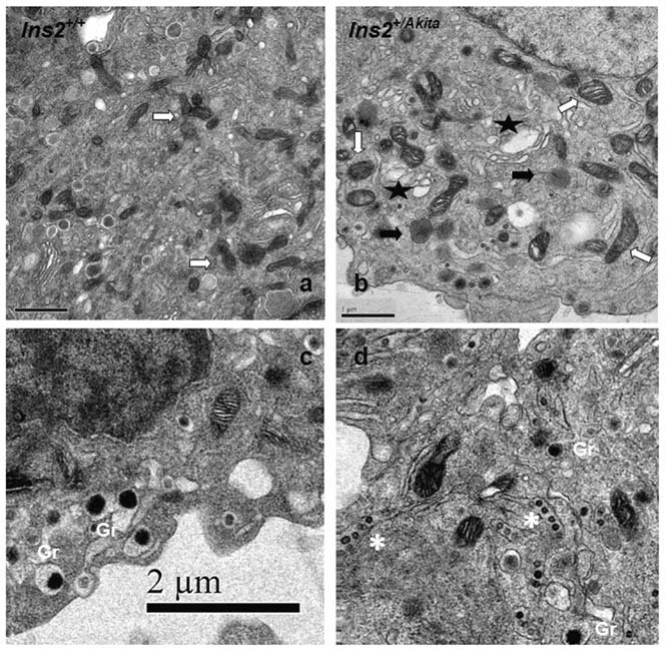
Structural abnormalities of *Ins2^+/Akita^* β-cells. We examined the subcellular structure of *Ins2^+/+^* (A and C) and *Ins2^+/Akita^* (B and D) β-cells after 48-hour customary culture by electric microscopy as described in the Materials and Methods. Asterisk, bean pod-like structure (that may represent atypically interacting forms of small insulin granules); Gr, mature insulin granules; white arrow, mitochondria; black arrow, lysosomes; 5-point star, enlarged endoplasmic reticulum (ER)/Golgi compartments or vacuoles; scale bar, 1 µm in (A and B) or 2 µm in (C and D). We performed quantitative evaluation of photographs from 10 β-cells for each group as described previously [42], and Table S1 presents the detailed data.

To verify the structural abnormalities preserved in the established *Ins2^+/Akita^* β-cells under *in vitro* culture maintenance, we performed electron microscopy examinations. Compared with controls, *Ins2^+/Akita^* β-cells demonstrated reduced numbers and size of mature insulin granules; enlarged ER, Golgi, and mitochondrion organelles; and increased numbers of lysosomes and vacuoles (Fig. 3; *P*<0.01, n = 10; see Table S1 for details). Intriguingly, a number of structures resembling bean pods appeared specifically in *Ins2^+/Akita^* β-cells that seemed to represent small atypically interacting insulin granules, but further investigation is required to clarify their nature. Because the *Ins2^+/Akita^* and *Ins2^+/+^* β-cells have been maintained at 25.5 mmol/L glucose since they were established in 2004 [30], these structural alterations evident in *Ins2^+/Akita^* β-cells and not control *Ins2^+/+^* β-cells clearly indicate that they are indeed toxic consequences of a primary PIHO disorder and do not result primarily from glucotoxicity *in vivo*.

### Severe oxidative stress in *Ins2^+/Akita^* β-cells/islets

**Figure 4:**
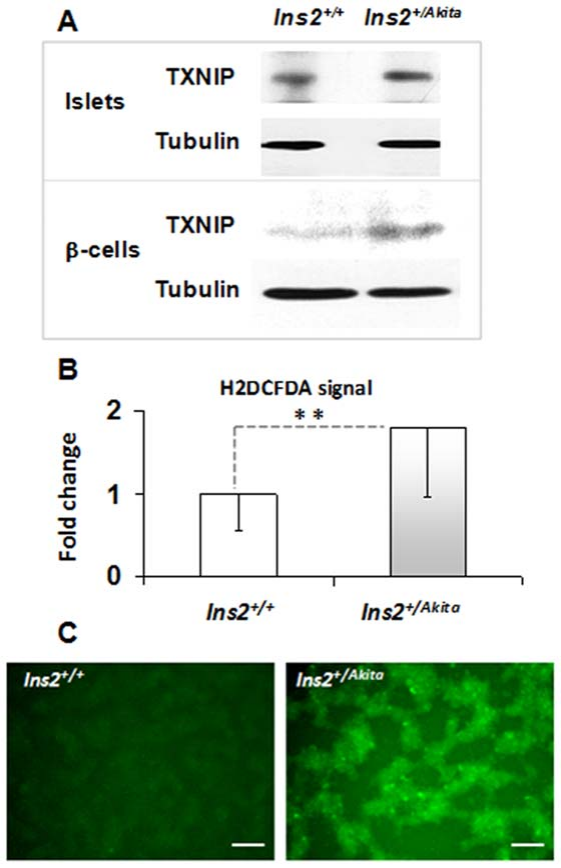
TXNIP and ROS increased in *Ins2^+/Akita^* β-cells/islets. (A) We performed immunoblot analysis of the levels of TXNIP in the *Ins2^+/Akita^* and *Ins2^+/+^* β-cells or islets (at 12 weeks of age). Protein amount per lane in (A), 40 µg. Fluorescent signals (C) and levels (B) of 2′,7′-dichlorodihydrofluorescein diacetate (H2DCFDA) fluorogenic marker (for ROS) in the established *Ins2^+/Akita^* and *Ins2^+/+^*β-cells. Scale bar in (C), 100 µm. Data are presented as the mean ± standard deviation (SD). n = 5; **, *P*<0.005.

**Figure 5:**
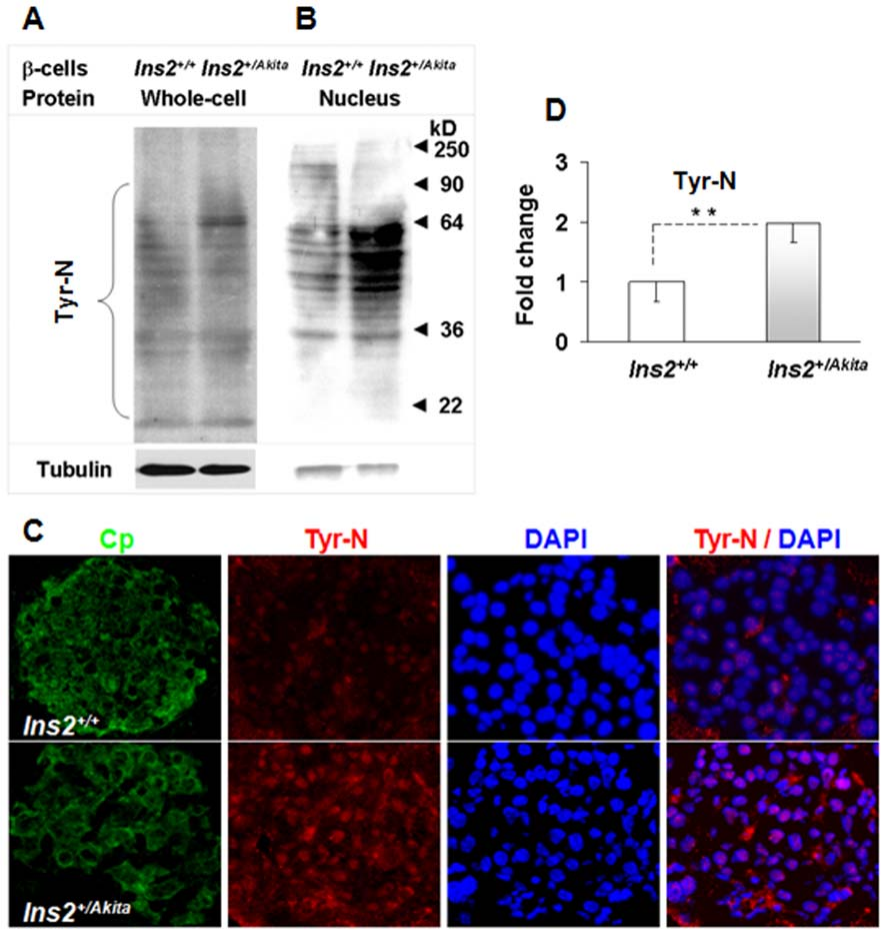
Protein tyrosine nitration (Tyr-N) enhanced in *Ins2^+/Akita^* β-cells/islets. We performed immunoblot analysis of the Tyr-N proteins in the protein pool of the entire cell (A) or its nucleus (B) of *Ins2^+/Akita^* and *Ins2^+/+^* β-cells. Amount of protein per lane in (A) or (B), 40 µg. C: We performed fluorescent staining to examine the immunoreactivities of Tyr-N protein (red color) in the β-cells that were marked with C-peptide (Cp) immunoreactivities (green color) and located within *Ins2^+/Akita^* (lower panels) or *Ins2^+/+^* (upper panels) islets at 12 weeks of age. Nuclei were marked by DAPI staining. D: The results show the nuclear abundance of Tyr-N proteins in the β-cells of *Ins2^+/Akita^* or *Ins2^+/+^* islets that were determined by using ImageJ software. Data in (D) are presented as the mean ± standard deviation (SD). n = 15; **, *P*<0.005.

Accumulating data indicate that the oxidative folding process of proteins in the ER generates ROS, and suboptimal folding conditions could increase ROS production [7]. We applied a number of oxidative stress markers to examine whether disorder in PIHO produces an increase in ROS in the established *Ins2^+/Akita^* β-cells and/or Akita islets. As shown in Figure 4A, the immunoreactive level of thioredoxin-interacting protein (TXNIP), a regulator of the cellular redox state [37], increased in both primary and established *Ins2^+/Akita^* β-cells. Likewise, the fluorescent signal of 2′,7′-dichlorodihydrofluorescein diacetate (H2DCFDA), a widely used reliable fluorogenic marker for ROS in live cells, increased significantly in the *Ins2^+/Akita^* β-cells compared to controls (Fig. 4B and C; fold, 1.8±0.9 versus 1.0±0.4; *P*<0.005, n = 5). We also examined the level of protein tyrosine nitration (Tyr-N) in *Ins2^+/Akita^* β-cells. In living cells, protein Tyr-N is generally mediated by ROS, such as peroxynitrite anion and nitrogen dioxide. Immunoblot analysis exposed an increase in the levels of Tyr-N proteins in *Ins2^+/Akita^* β-cells (Fig. 5A), which was more apparent in the nucleus protein pool (Fig. 5B). In histological studies, Tyr-N protein immunoreactivity was predominant in the nuclei of the β-cells of 12-week-old *Ins2^+/Akita^* islets and higher than in control β-cells of 12-week-old *Ins2^+/+^*islets (Fig. 5C and D; fold, 2.0±0.2 versus 1.0±0.3; *P*<0.005, n = 15). These findings clearly demonstrate the occurrence of severe oxidative stress in both primary and cloned *Ins2^+/Akita^* β-cells.

### Altered death and proliferation rates of *Ins2^+/Akita^* β-cells

**Figure 6:**
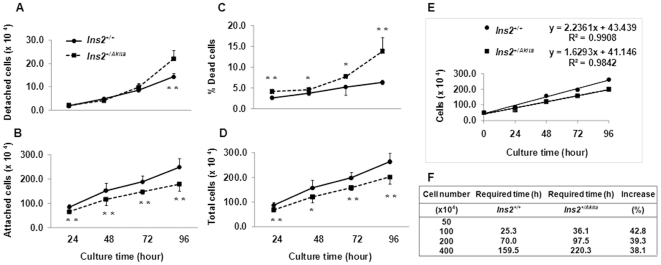
Altered death and proliferation rates of *Ins2^+/Akita^* β-cells. At the indicated time-points after *Ins2^+/Akita^* and *Ins2^+/+^*β-cells with the same passage numbers were seeded on 6-well/plates (5.0×10^5^ cells/well) and cultured in the customary medium, we examined the number of detached (A), attached (B), and dead *Ins2^+/Akita^* and *Ins2^+/+^*β-cells using the approach described in Materials and Methods. We calculated the percentage of dead cells (C) in the total *Ins2^+/Akita^* or *Ins2^+/+^*β-cells at each of the indicated time points (D) using the formula: dead cell (%) = (dead cell number)×100%/(dead cell number+alive cell number). Data in (A to D) are presented as the mean ± standard deviation (SD). n = 6. *, *P*<0.05; **, *P*<0.005. (E) Based on the total cell numbers examined at individual time-points, we obtained 2 reliable formulas to estimate the numbers (Y) of *Ins2^+/+^* or *Ins2^+/Akita^* β-cells at a given time-point (X, 24≤X≤96, hours). The nearer the R-squared value (relative predictive power of a formula model) to one, the better the model. (F) We used the formulas in (E) to calculate the times (hours) required for the initially seeded 5.0×10^5^ *Ins2^+/+^* or *Ins2^+/Akita^* β-cells/well to propagate up to the numbers listed in the first column. The increase (%) refers to the increased percentage in the hours required for the numbers of *Ins2^+/Akita^* versus *Ins2^+/+^* β-cells to double.

To clarify whether PIHO disorder alters the rates of death and proliferation of β-cells, we compared these rates in *Ins2^+/Akita^* and *Ins2^+/+^*β-cells. The rates were assessed by the number/percentage of alive and dead cells during culture periods that was determined by trypan blue staining procedure. This procedure that detects dead cells via apoptotic and/or necrotic pathways is described in the Materials and Methods and Figure 6 legend. At 24, 48, 72, and 96 hours after we seeded cells (on 6-well plates) for culture under the customary condition, we observed a significant increase in the number of detached *Ins2^+/Akita^* β-cells only at 96 hours (Fig. 6A, *P*<0.01, n = 6). Staining with trypan blue dye indicated that all detached cells were dead (not shown), which suggests that the detached dead *Ins2^+/Akita^* β-cells did not increase until the end of the 4-day observation period. However, the numbers of *Ins2^+/Akita^* β-cells attached (on culture plates) or attached and detached (total number) decreased significantly (*P*<0.05, n = 6) at each examination time-point from the beginning (Fig. 6B and D). The decrease in the total number of *Ins2^+/Akita^* β-cells at the individual examination time-points resulted partially from an increase in the percentage of dead cells (i.e., detached and somewhat attached) compared to the proportion in control *Ins2^+/+^* β-cells (Fig. 6C; 24 hours, 4.1±0.5 versus 2.6±0.4; *P*<0.0001; 48 hours, 4.5±0.6 versus 3.7±0.4, *P*<0.05; 72 hours, 7.7±0.6 versus 5.3±1.9, *P*<0.05; 96 hours, 13.8±3.3 versus 6.3±0.5, *P*<0.001; n = 6).

Based on the total numbers of cells examined at the individual time-points, we obtained 2 reliable formulas to estimate the times required for *Ins2^+/+^* or *Ins2^+/Akita^* β-cells to double their numbers under customary culture conditions (Fig. 6E, see detailed explanations in the figure legend). The times required to double the number of *Ins2^+/Akita^* β-cells increased 38.1 to 42.8% compared with the doubling time of control *Ins2^+/+^* β-cells (Fig. 6F). The less than 3% difference between percentages of dead cells in these 2 β-cell lines (with the same passage numbers) at all time-points except 96 hours (Fig. 6C) cannot fully explain this 4.7% increase in the hours required for a cycle of cells to double in number. These results suggest that decelerated proliferation of *Ins2^+/Akita^* β-cells may contribute to the large differences between the total numbers of cells in these 2 β-cell lines at examination time-points (Fig. 6D). This proposition was proven true because *Ins2^+/Akita^* β-cells have slower proliferation rates than control *Ins2^+/+^*β-cells in 5′-bromo-2′-deoxyuridine (BrdU) labeling assays (Fig. S2, *P*<0.005; n = 3).

## Discussion

To understand better the defects that result from a primary PIHO disorder, we further investigated model *Ins2^+/Akita^* β-cells/islets. First, quantitative PCR, metabolic-labeling, and immunoblotting studies (Fig. 1) showed no apparent defect in the contribution by proinsulin transcription or translation to the reduction of cellular proinsulin and insulin in *Ins2^+/Akita^* β-cells. Because we maintained *Ins2^+/Akita^* and *Ins2^+/+^* β-cells under the same culture conditions, our results excluded contributions from possible differences in glucose effect and/or β-cell population that sometimes appear in studies using islets of diabetic versus normal subjects. These data thus supported the primary responsibility of abnormalities in the early post-translational processing of insulin precursor for reduced proinsulin and insulin content and secreted insulin in Akita mice [31]–[33]. They do not support the enhancement of eukaryotic initiation factor 2 (eIF2α) phosphorylation for translational attenuation [7] as the principal protective mechanism in β-cells with PIHO disorders, although this pathway is active in many dysfunctional cell types. The reason is that no noticeable attenuation of proinsulin or whole protein biosynthesis was found in primary and cloned *Ins2^+/Akita^* β-cells (Fig. 1C) [30]–[33]. Moreover, excessive eIF2α phosphorylation is poorly tolerated by human islets and exacerbates apoptosis induced by fatty acids [38].

Second, the immunoblot result (Fig. 2) provides the first clear evidence of the responsibility of a primary disorder in PIHO for impaired GSIS at 15 or 120 minutes, although the response of proinsulin biosynthesis to glucose stimulation was customarily maintained in *Ins2^+/Akita^* β-cells. These results also confirmed a recent finding of a primary PIHO disorder causing a disproportionally high ratio of proinsulin/insulin in the secreted proteins of *Ins2^+/Akita^* islets/β-cells [32]. Although mechanistic models for GSIS or proinsulin conversion to insulin in normal β-cells have been well established [35], [39], the mechanisms underlying impaired GSIS in type 1 or type 2 diabetes remain largely unclear [34], [36]. The findings shown in Figure 2 underscore a potentially important role of PIHO disorder in the impaired GSIS and disproportionate hyperproinsulinemia in diabetes.

Third, histological studies (Fig. 3, Table S1) disclosed that various structural abnormalities preserved by the established *Ins2^+/Akita^* β-cells, including reduced size and number of mature insulin granules, enlarged ER, Golgi, and mitochondrion organelles, and increased numbers of lysosome and vacuoles, were comparable to abnormalities observed in Akita islets [31], [33], [40]–[42]. The data excluded the contribution from glucotoxicity because *Ins2^+/+^* β-cells did not develop such abnormalities, although both *Ins2^+/Akita^* and *Ins2^+/+^* β-cells were cultured at the same high glucose condition from the time they were established [30]. On the one hand, the reduced numbers and size of mature insulin granules caused by PIHO disorders should be a basis for the impaired GSIS of *Ins2^+/Akita^* β-cells. On the other, PIHO disorder would yield enlarged/increased early secretory compartments, energy supply, and protein disposal apparatuses as adaptations or consequences. Alterations, such as the lessening (or degranulation) of mature insulin granules and expansion of varied organelles, are frequently noticeable in animal models and humans with diabetes induced by various factors [43]–[47]. The similarity in structural abnormalities supports the suggestion that the genetic and stress-susceptible PIHO disorder may be a general contributor in the structural defect of β-cells in diabetes.

Fourth, although a link is recognized between ER stress (as a consequence of misfolded proteins) and oxidative stress [7], our investigation showed the first clear evidence that PIHO disorder produces severe oxidative stress, manifested by a significant increase in the ROS, TXNIP, and protein Tyr-N levels of *Ins2^+/Akita^* β-cells/islets (Figs. 4 and 5). It is known that oxidoreductases, such as ER oxidoreductin 1 (ERO1) and protein disulfide isomerase (PDI), generally drive the thiol-disulfide equilibrium toward disulfide bond formation of proteins in the ER. PDI directly catalyzes the disulfide bond formation in nascent proteins and transfers electrons from thiols to ERO1. ERO1 passes the electrons to molecular oxygen and generates ROS in the process [7]. Correct conformation of proinsulin, which requires formation of 3 intramolecular disulfide bonds with assistance of members of the ERO1 and PDI families, can generate ROS. Disorder in PIHO could cause repeated unproductive cycles of proinsulin oxidation and reduction and increased ROS production from the ER, if sustained, result in calcium leakage from the ER lumen and uptake into the mitochondrial matrix, which would enhance production of ROS also in the mitochondria as that observed in other cell types with induced ER dysfunction [48].

Intriguingly, the increase of protein Tyr-N seems to be predominant in the nucleus of *Ins2^+/Akita^* β-cells/islets (Fig. 5), which suggests that the protein Tyr-N is highly active in the nuclei of β-cells with PIHO disorders. Although no data has been produced to date regarding how the enhanced aggregation and disposal of proinsulin is associated with this kind of oxidative stress, recent progress in biomedical research provides clues to our understanding of the finding. As recognized in the consequences of protein misfolding, ROS increase is observed in *Ins2^+/Akita^* β-cells that preserve an enhanced proinsulin aggregation and disposal as a result of a point mutation in the *Ins2* gene. Increased ROS such as free radical superoxide and/or hydrogen peroxide may facilitate nuclear translocation of some proteins such as the transcriptional factor NF-κB and lead to the formation of peroxynitrite, a molecule that is a powerful oxidant [2], [6], [49]. Because of its oxidizing and membrane-penetrating properties, peroxynitrite can damage or modify various molecules in cells, including DNA and proteins. Tyr-N is known as a main mechanism that underlies protein damages/modifications provoked by peroxynitrite anion and other ROS such as nitrogen oxide. As reviewed [50], Tyr-N occurred in proteins such as the NF-κB, iκBα, PPARγ, p53, and histones that are predominantly located in the nucleus of cells. In *Ins2^+/Akita^* β-cells/islets, some of these processes that would possibly occur may contribute to the abundant nuclear localization of Tyr-N proteins observed in this study. Furthermore, the increase of signals of H2DCFDA and TXNIP in addition to protein Tyr-N (Fig. 4) indicates insult of the entire cell and not just the ER compartments by oxidative stress in *Ins2^+/Akita^* β-cells. These results suggest that the genetic and stress-susceptible PIHO disorder may contribute to oxidative stress manifested as increased ROS (e.g., hydrogen peroxide) and/or protein Tyr-N, markers well demonstrated in dysfunctional β-cells in type 1 [1], [2], [51] or type 2 diabetes [2], [6], [7], [46], [47].

Lastly, our studies provide the first demonstration that PIHO disorder concurrently augments death and decelerates proliferation of β-cells (Figs. 6 and S2). Because we subjected *Ins2^+/Akita^* β-cells and control *Ins2^+/+^* β-cells with identical passage numbers to the same experimental procedure under *in vitro* culture condition, this data excluded the contributions from possible divergences in glucose concentration, β-cell number, and/or *in vivo* neogenesis that may occur in studies using islets of subjects with hyperglycemia versus euglycemia. Thus, the simultaneous augmentation of cell death and deceleration in cell proliferation are indeed a consequence of PIHO disorder in this dysfunctional β-cell model. This demonstration aids understanding of discrepancies in the examined *in vivo* β-cell death in Akita mice [33], [52] and reveals the decelerated proliferation that would contribute to progressive loss of β-cells in Akita mice. Although unfolded protein responses, such as the Chop-10/gadd153 pathway known to be implicated in the increased death of *Ins2^+/Akita^* β-cells [52], are activated by PIHO disorders, the mechanisms underlying the decelerated replication remain unclear. In diabetes of type 1 and type 2, the increase in β-cell apoptosis is known to contribute to β-cell depletion as the disease develops [2], [5], [10], but it remains unclear whether β-cell proliferative responses contribute to the increase [10]. A recent study shows decreased β-cell proliferation with type 2 diabetes in obese donors [45]. The decelerated replication of β-cells as a consequence of PIHO disorders may be associated with a deficit in β-cell mass in diabetes.

In summary, our findings provide the first clear demonstration that perturbation of PIHO produces severe oxidative stress and impairs GSIS and β-cell survival. In model *Ins2^+/Akita^* β-cells/islets, the anomalies we characterize represent the extensive defects that arise from PIHO disorder and are known to be associated with diabetes, including insulin deficiency, GSIS impairment, organelle structure abnormality, oxidative and ER stress, decelerated cell proliferation, increased cell death, disproportionally high proinsulin/insulin ratio [32], and insulin resistance [53]. However, disorders in PIHO occur in several unique diabetic models with mono/poly-genetic defects that represent the primary forms of diabetes in humans (unpublished data). PIHO is susceptible to genetic and environmental influences, and its susceptibility is linked to the inherent low relative folding rate versus plentiful amount of insulin precursor manufactured in β-cells [23]. Accordingly, PIHO disorder would contribute to β-cell defects in diabetes of general forms in addition to monogenic diabetes with heterogeneous mutations in the insulin gene. Unquestionably, the properties and extent of PIHO disorder, duration of development of β-cell failure, and symptoms of diabetes may vary greatly because of differences in inducers. An example is the diverse symptoms of patients with heterogeneous mutations in the same preproinsulin molecule [26]–[30]. Thus, the molecular mechanisms underlying the maintenance, perturbation, and consequences of PIHO deserve further study to develop approaches aimed at preserving or restoring β-cell function in diabetes.

## Materials and Methods

### Ethics statement

All animal and tissue sample experiments have been approved by the Institutional Animal Care and Use Committee of The Ohio State University (protocol number 2007A0040 and 2010A0024) and were performed in accordance with the guidelines of the National Institutes of Health and The Ohio State University.

### Reagents, cell lines, and mice

In this study, we applied antibodies against rat C-peptide II (Millipore, Bedford, MA, USA), insulin (Dako North America, Inc.), TXNIP (Santa Cruz Biotechnology, Inc., Santa Cruz, CA, USA), tubulin (Sigma-Aldrich, St. Louis, Mi, USA), and tyrosine-nitrated proteins (kindly provided by Dr. J.A. Hinson, University of Arkansas for Medical Sciences, Little Rock, AR, USA) [54]. Second antibodies were purchased from Jackson ImmunoResearch Laboratories (West Grove, PA, USA). We obtained proinsulin, dithiothreitol, collagenase, and 5′-bromo-2′-deoxyuridine (BrdU) from Sigma-Aldrich; TRIZOL and reverse transcription (RT)-PCR reagents, H2DCFDA, culture media from Invitrogen (Carlsbad, CA, USA); 4′,6-diamidino-2-phenylindole (DAPI) and protease inhibitor cocktail from Roche Applied Science (Indianapolis, IN, USA). We obtained Immobilon-P^SQ^ membrane from Millipore, and [^35^S]-methionine (3.7×10^13^ Bq/mmol) from Perkin Elmer Life Science (Waltham, MA, USA). The *Ins2^+/+^* and *Ins2^+/Akita^* β-cell lines were kindly provided by Drs. H. Kubota and K. Nagata (Kyoto University, Kyoto, Japan). The C57BL/6J and *Ins2^+/Akita^* mice were purchased from The Jackson Laboratory (Bar Harbor, Maine, USA).

### Islet preparation and cell culture

Islet isolation and culture of islets in 10% FCS/RPMI 1640 medium (containing 11 mmol/L glucose) and established *Ins2^+/+^* or *Ins2^+/Akita^* β-cells in the customary10% FCS/DMEM medium (containing 25.5 mmol/L glucose if no noted specifically) were described previously [17], [30]–[32].

### Semi-quantitative PCR

The procedure was described previously [31]. Briefly, RNAs of the established *Ins2^+/+^* and *Ins2^+/Akita^* β-cells were extracted by using TRIZOL reagent for reverse transcription. Semi-quantitative examination of insulin transcripts were performed by PCR amplification with a set of primers derived from the identical region of insulin 1 and insulin 2 genes. The sequence of primers for insulin is 5′-GCTCTCTACCTGGTGTGTGG-3′ and 5′-GTTTTATTCATTGCAGAGGG-3′ and for β-actin is 5′-CGTAAAGACCTCTATGCCAA-3′ and 5′-AGCCATGCCAATGTTGTCTC-3′ [31]. The expected size of PCR product was 257 bp for insulin1, 263 bp for insulin 2, and 349 bp for β-actin.

### Metabolic-labeling and autoradiography

*Ins2^+/+^* and *Ins2^+/Akita^* β-cells were cultured in the customary 10% FCS/DMEM medium for 48 h and then labeled with [^35^S]-methionine for 30 min as described previously [17], [23], [31]. Cellular proteins were then subjected to immunoprecipitation with C-peptide antisera. Immunoprecipitates were resolved by reduced tricine sodium dodecyl sulfate polyacrylamide gel electrophoresis (SDS-PAGE) for autoradiography as described previously [23], [31].

### Immunoblotting

Whole-cell, nucleus, or secreted proteins were resolved by SDS-PAGE for immunoblot analyses as described previously [23], [31]. In this study, reduced SDS-PAGE was applied (except for the non-reduced SDS-PAGE used only in Fig. 2B). For collection of cell nucleus fraction, cells were homogenized in a homogenate buffer in a glass, hand driven tissue homogenizer and then subjected to centrifugation with a centrifugal force of 600 g for 15 min at 4°C as described previously [17].

### Histological studies

*Ins2^+/+^* and *Ins2^+/Akita^* β-cells were cultured in the customary 10% FCS/DMEM medium for 48 h and their light microscopic images were then examined with an Axiovert 200 microscope (Carl Zeiss, Oberkochen, Germany). Fluorescent staining with DAPI and antibodies against to tyrosine-nitrated proteins (1∶500) [54] and C-peptide (1∶500) on pancreatic sections (5 µm thickness) was performed as described previously [17], [31]. Fluorescent images were examined under Axiovert 200 microscope with Axiovision software.

Electron microscopic examination approaches were described previously [17], [31]. Briefly, cells were fixed with 2% glutaraldehyde and paraformaldehyde in 100 mmol/L sodium cacodylate buffer (pH 7.4) for 2 h, incubated in 1% osmium tetroxide in 100 mmol/L sodium cacodylate buffer for 1 h, stained with 1% uranyl acetate in maleate buffer (pH 5.1) for 1 h, and then dehydrated through a graded ethanol series. After infiltration and polymerization in a propylene oxide and spurr resin mixture, the embedded samples were cut with a Reichert Ultracut-E microtome (Reichert-Jung, Vienna, Austria), and stained with uranyl acetate and lead citrate. Images were observed with a FEI Tecnai F30 electron microscope at accelerating potential of 300 kV. The quantitative evaluation of photographs from 20 β-cells each group was performed as described previously [42].

### Examination of reactive oxygen species

After 48 h culture in the customary 10% FCS/DMEM medium, *Ins2^+/+^* and *Ins2^+/Akita^* β-cells were treated with 5 µmol/L H2DCFDA for 10 min according to the Instructions (Invitrogen). H2DCFDA fluorescent images were examined under Axiovert 200 microscope with Axiovision software.

### Examination of dead and alive cell numbers and cell proliferation

*Ins2^+/Akita^* and *Ins2^+/+^*β-cells with same passage numbers were seeded on 6-well/plates (5.0×10^5^ cells/well) and cultured in the customary 10% FCS/DMEM medium. At 24, 48, 72, and 96 h after cells were seeded, detached cells (in media) and attached cells (on plates) were harvested. Dead or alive cells were determined by trypan blue (Invitrogen) staining method according to the manufacturer's instructions and then counted in haemocytometer chambers. For measurement of cell proliferation by BrdU labeling assay, *Ins2^+/Akita^* and *Ins2^+/+^*β-cells after seeded for one day were incubated with 10 mmol/L BrdU for 24 h. Cells were fixed and subjected to staining with DAPI and anti-BrdU (1∶200, Sigma-Aldrich) antibody as described previously [17], [31]. Proliferative index is expressed as a percentage of BrdU- and DAPI-positive cells over total DAPI -positive cells analyzed under Axiovert 200 microscope with Axiovision software.

### Statistical analysis

Densitometry of fluorescent images was quantified by NIH ImageJ software. Data are shown as the mean or the mean ± SD. Statistical significance (*P<0.05; **P<0.01) was assessed by Student's *t*-test (two-tailed). All experiments were performed at least three independent times.

### Supplemental Data

Supplemental Data, including 2 figures and 1 table, can be found with this article on the *PLoS ONE* web site.

## Supporting Information

Figure S1 **Levels of secreted insulin and proinsulin that were immunopurified by insulin antisera or mixed antisera to insulin and C-peptide.** (**A**) Proinsulin and insulin secreted by *Ins2^+/+^* β-cells during a 120-minute customary glucose (25.5 mmol/L) culture course were immunopurified with insulin (Ins) antisera or mixed antisera to insulin and C-peptide (Cp), resolved with proinsulin marker (Sigma) by 16.5% tricine non-reduced SDS-PAGE, and examined by insulin immunoblot. (B) The relative levels of proinsulin or insulin immunoprecipitated by Ins or Ins combined with Cp antisera shown in (A). Marker, proinsulin marker (Sigma); IP, immunoprecipitation; n = 3; *, *P*<0.05.
(TIF)

Figure S2 **Decelerated proliferation of** ***Ins2^+/Akita^*** **β-cells.** *Ins2^+/Akita^* and *Ins2^+/+^*β-cells after one day culture were then incubated with BrdU (10 mmol/L) in the customary culture medium for 24 h. Proliferative index was determined as the number of BrdU- and DAPI-positive cells over total DAPI-positive cells, and data are presented as the percentage of BrdU- and DAPI-positive cells in the total DAPI-positive cells. n = 3; **, *P*<0.005.
(TIF)

Table S1 **Morphometric analysis of** ***Ins2^+/+^*** **versus** ***Ins2^+/Akita^*** **β-cells.**
(PDF)
