# Supplementary figures and images for: Proinsulin Atypical Maturation and Disposal Induces Extensive Defects in Mouse Ins2+/Akita β-Cells
Source: PLoS One. 2012 Apr 3;7(4):e35098. doi: 10.1371/journal.pone.0035098 (PMC3318013; doi:10.1371/journal.pone.0035098)

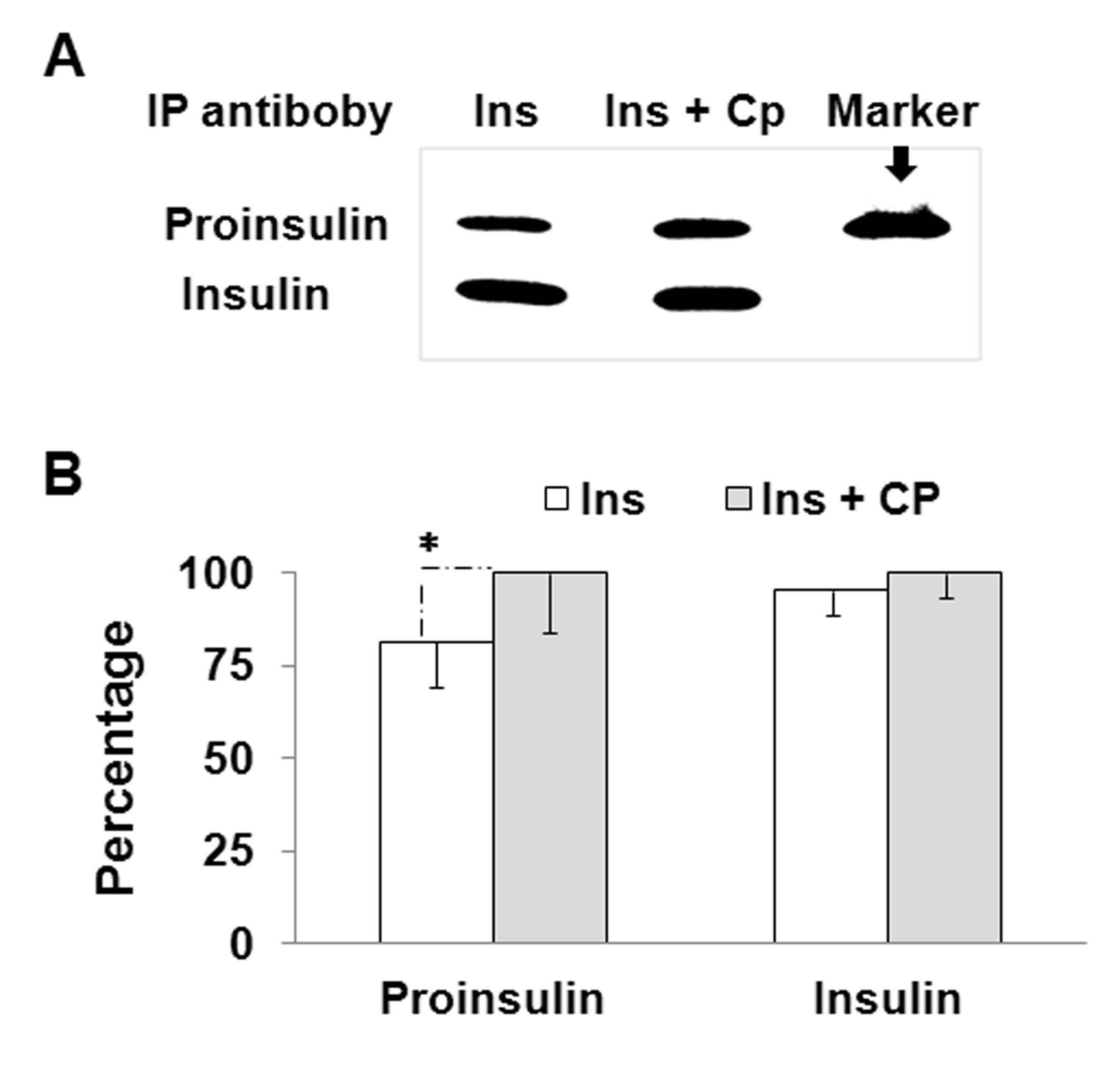

Supplement: Figure S1 — Levels of secreted insulin and proinsulin that were immunopurified by insulin antisera or mixed antisera to insulin and C-peptide. (A) Proinsulin and insulin secreted by Ins2+/+ β-cells during a 120-minute customary glucose (25.5 mmol/L) culture course were immunopurified with insulin (Ins) antisera or mixed antisera to insulin and C-peptide (Cp), resolved with proinsulin marker (Sigma) by 16.5% tricine non-reduced SDS-PAGE, and examined by insulin immunoblot. (B) The relative levels of proinsulin or insulin immunoprecipitated by Ins or Ins combined with Cp antisera shown in (A). Marker, proinsulin marker (Sigma); IP, immunoprecipitation; n = 3; *, P<0.05. (TIF) [file pone.0035098.s001.tif]

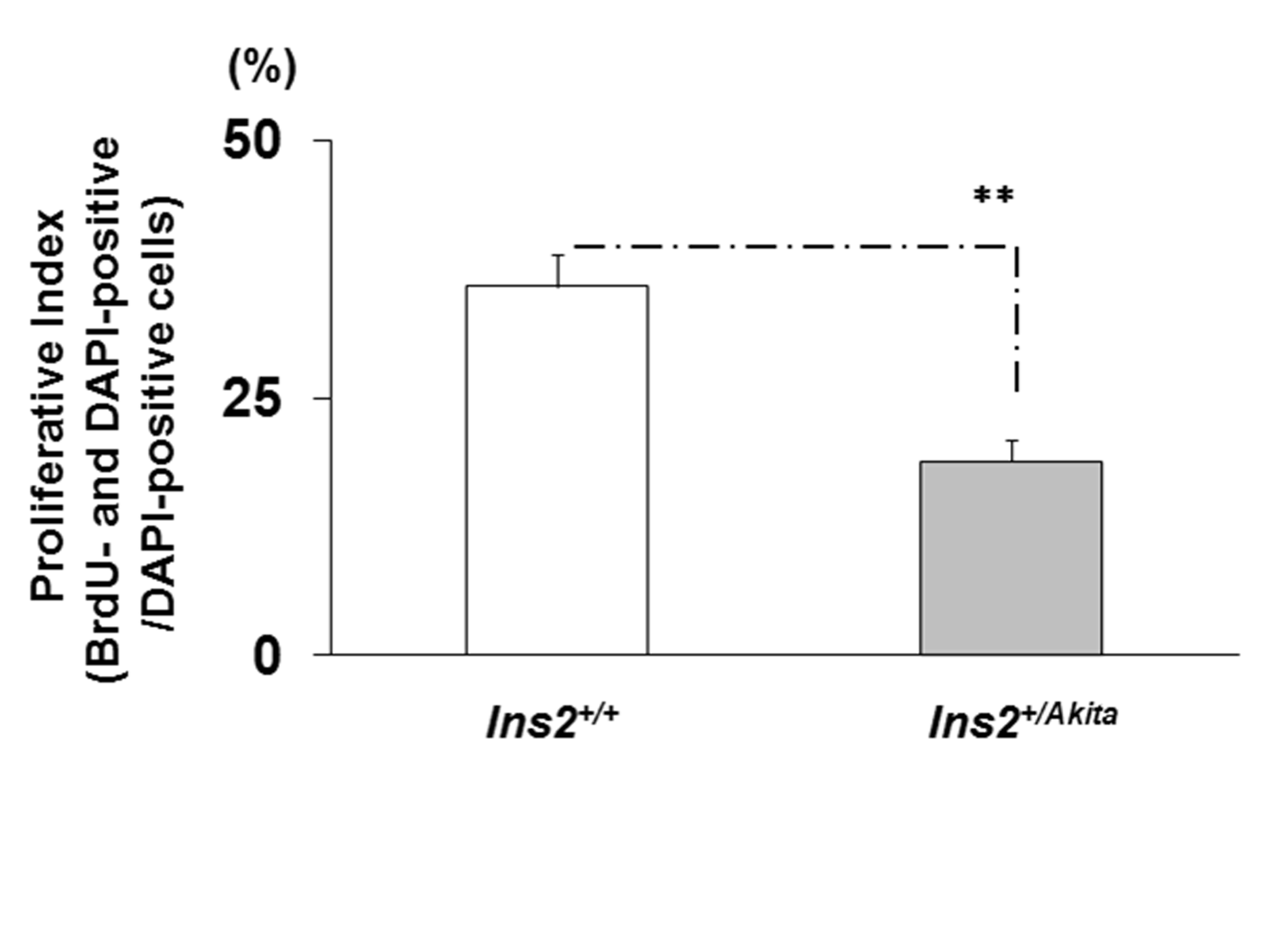

Supplement: Figure S2 — Decelerated proliferation of Ins2+/Akita β-cells. Ins2+/Akita and Ins2+/+β-cells after one day culture were then incubated with BrdU (10 mmol/L) in the customary culture medium for 24 h. Proliferative index was determined as the number of BrdU- and DAPI-positive cells over total DAPI-positive cells, and data are presented as the percentage of BrdU- and DAPI-positive cells in the total DAPI-positive cells. n = 3; **, P<0.005. (TIF) [file pone.0035098.s002.tif]
