# Supplementary material for: Proinsulin Atypical Maturation and Disposal Induces Extensive Defects in Mouse Ins2+/Akita β-Cells
Source: PLoS One. 2012 Apr 3;7(4):e35098. doi: 10.1371/journal.pone.0035098 (PMC3318013; doi:10.1371/journal.pone.0035098)
Supplement: Table S1 — Morphometric analysis of Ins2+/+ versus Ins2+/Akita β-cells. (PDF) [file pone.0035098.s003.pdf]

Table S1. Morphometric analysis of established *Ins2*$^{+/Akita}$ and *Ins2*$^{+/+}$ β-cells

	Ins2$^{+/+}$	*Ins2*$^{+/Akita}$	*P*
**ER**	100.0 ± 13.7	196.8 ± 20.3	< 0.001
**Golgi**	100.0 ± 16.8	385.3 ± 39.1	< 0.001
**Size of insulin granules**	100.0 ± 11.9	68.2 ± 5.3	< 0.01
**Number of insulin granules**	100.0 ± 9.2	51.3 ± 4.6	< 0.001
**Number of lysosomes**	100.0 ± 8.8	203.3 ± 16.2	< 0.001
**Number of vacuoles**	100.0 ± 12.4	325.1 ± 29.1	< 0.01
**Size of mitochondron**	100.0 ± 12.5	159.1 ± 15.9	< 0.01

Quantitative evaluation of photographs from 10 β-cells for each group was performed as described previously (42) and data are shown.
